# Antibody-Dependent Enhancement of Dengue Virus Infection in Primary Human Macrophages; Balancing Higher Fusion against Antiviral Responses

**DOI:** 10.1038/srep29201

**Published:** 2016-07-06

**Authors:** Jacky Flipse, Mayra A. Diosa-Toro, Tabitha E. Hoornweg, Denise P. I. van de Pol, Silvio Urcuqui-Inchima, Jolanda M. Smit

**Affiliations:** 1 Department of Medical Microbiology, University Medical Center Groningen, University of Groningen, the Netherlands; 2 Grupo Immunovirología, Facultad de Medicina, Universidad de Antioquia UdeA, Medellín, Colombia

## Abstract

The dogma is that the human immune system protects us against pathogens. Yet, several viruses, like dengue virus, antagonize the hosts’ antibodies to enhance their viral load and disease severity; a phenomenon called antibody-dependent enhancement of infection. This study offers novel insights in the molecular mechanism of antibody-mediated enhancement (ADE) of dengue virus infection in primary human macrophages. No differences were observed in the number of bound and internalized DENV particles following infection in the absence and presence of enhancing concentrations of antibodies. Yet, we did find an increase in membrane fusion activity during ADE of DENV infection. The higher fusion activity is coupled to a low antiviral response early in infection and subsequently a higher infection efficiency. Apparently, subtle enhancements early in the viral life cycle cascades into strong effects on infection, virus production and immune response. Importantly, and in contrast to other studies, the antibody-opsonized virus particles do not trigger immune suppression and remain sensitive to interferon. Additionally, this study gives insight in how human macrophages interact and respond to viral infections and the tight regulation thereof under various conditions of infection.

Through the evolutionary process, viruses have acquired many mechanisms to hijack the host cell machinery and to suppress antiviral responses within infected cells. More intriguingly, viruses have found ways to antagonize the host immune system by using the hosts’ antibodies to enhance infection and disease: a phenomenon called antibody-dependent enhancement (ADE)^1^,^2^. *In vitro,* ADE has been shown for influenza A virus^3^, Coxsackievirus B^4^, respiratory syncytial virus^5^, Ebola virus^6^, human immunodeficiency virus (HIV)^7^, and many other viruses^8^. *In vivo* ADE has been linked with the severity of HIV^7^ and dengue virus (DENV) infection^9^,^10^.

DENV infection is the most prevalent arthropod-borne viral infection worldwide with approximately 390 million infections and 96 million symptomatic cases in 2010^11^. Four serotypes of DENV exist (DENV1–4) and symptomatic infection with any DENV serotype leads to mild dengue fever or to the life-threatening dengue haemorrhagic fever and dengue shock syndrome^9^.

The question why some patients develop dengue fever, and others dengue haemorrhagic fever or dengue shock syndrome is continuously under investigation and debate. Epidemiologic research showed that severe dengue disease is strongly associated with primary infection of infants with waning maternal anti-dengue immunity^10^,^12^, and with secondary, heterotypic dengue infection^13^,^14^,^15^,^16^. Patients with severe dengue disease often present high viral loads early in infection^17^,^18^,^19^. In line with this, heterotypic sub-neutralizing antibodies, or waning concentrations of homotypic antibodies have been found to enhance DENV infectivity *in vitro* and *in vivo*^12^,^20^,^21^.

So far, little is known about how antibodies enhance DENV infection and disease^20^,^22^. Previously, antibodies were found to increase the number of infected cells and, subsequently, facilitate higher virus production once the concentration of the antibody falls below the neutralization threshold^23^,^24^,^25^,^26^. Furthermore, it has been suggested that DENV immune-complexes would boost virus production per infected cell (burst size) by suppressing intracellular antiviral responses^22^,^27^,^28^,^29^. The latter is also called *intrinsic* ADE to stress putative involvement of an intracellular mechanism. Consequently, enhancement of the infected cell mass is named *extrinsic* ADE.

DENV replicates in macrophages, monocytes, and dendritic cells *in vitro*^24^,^30^,^31^ and *in vivo*^32^. During secondary infection, *i.e.* in the presence of antibodies, monocytes and macrophages actively support ADE, whereas immature dendritic cells do not^23^,^24^,^33^. Indeed, studies with primary macrophages and monocytes found that ADE can enhance DENV burst sizes in the order of 5.3- to 7.2-fold^23^,^24^,^25^. Here, we focused on one cell type, i.e. primary human macrophages, and attempted to discern the mechanism of enhanced DENV infection within this cell type.

We discovered that, in primary human macrophages, antibody-mediated cell entry of DENV enhances the fusion potential of the virus. No enhanced binding and entry was seen. Also, we observed that ADE does not induce an increased antiviral response early in infection. We propose that the increase in membrane fusion activity initiates an aggravating cascade leading to the typical enhancement of (i) infection, (ii) burst size, and (iii) disproportionally stronger anti-inflammatory responses. Importantly, no antibody-mediated immunosuppressive signalling was detected in primary human macrophages. Our data suggests that DENV antagonizes the host antibody as a vehicle to enhance its fusion efficiency within macrophages and consequently enhance disease severity.

## Methods

### Antibodies

Human monoclonal antibodies (#753 C6 and #751 A2) against the DENV2 E protein were a kind gift of J. Mongkolsapaya and G. Screaton (Imperial College, UK). Human IgG Fc-fragments were used as control (Jackson immunoResearch, USA). Antibodies for the phenotyping of the macrophages, and corresponding isotypes, were obtained through an unrestricted grant (Immunotools, DE).

### Cell lines

Baby Hamster Kidney cells (BHK-15), gift of Richard Kuhn, Purdue University, were propagated in Dulbecco’s minimal essential medium (DMEM) (Gibco, NL) supplemented with 10% Fetal Bovine Serum (FBS) (Lonza, USA), 100 U/mL penicillin and 100 mg/mL streptomycin (PAA, Switzerland), 0.75 g/L sodium bicarbonate (Gibco). The green monkey-derived Vero WHO cell line (WHO RCB 10–87), gift of James Brien, was cultured as described for the BHK-15, yet at 5% FBS. P338D1 cells (ATCC, #CCL-46) were cultured as described for BHK-15, with addition of 1mM sodium pyruvate (Gibco).

### Macrophages

Buffy coats were obtained from anonymous donors with informed consent from Sanquin blood bank (Groningen, the Netherlands), in line with the declaration of Helsinki. The flavivirus-immune status of the 23 blood donors that we used is unknown but it is unlikely that a major proportion is flavivirus-positive. PBMCs were isolated by Ficoll-Paque (GE Healthcare, Belgium) gradient, and stored in 90% FBS, 10% DMSO on liquid nitrogen till use. Monocytes were isolated from total PBMCs, as described previously^31^, and differentiated into macrophages by culturing in 12-wells plates for 6 days at 37 °C, 5% CO_2_ in RPMI with HEPES (Gibco), supplemented with 20% FBS and 100 ng/mL recombinant human M-CSF (Prospec-Tany, Israel). On alternate days, 75% of the medium was replaced with medium and the full amount of M-CSF.

### Virus stocks

Dengue virus serotype 2, strain 16681 (DENV2) was propagated on C6/36 cell line, as described previously^34^,^35^. The specific infectivity of the virus stock was 79 genomes per PFU. Purification was performed as described by Ayala *et al*.^35^. Vesicular Stomatitis Virus, Indiana serotype, strain San Juan A (VSV) was propagated on Vero WHO cells. Vero WHO cells were infected at MOI 0.1 in cell culture medium with 2% FBS. After 24 h, the culture medium was harvested, clarified, and mixed with HEPES (PAA, Switzerland) to 10 mM final concentration. All virus samples were aliquoted and snap frozen in liquid nitrogen prior to storage at −80 °C.

Infectious virus titers were determined by plaque assay. DENV2 titers were determined on BHK-15 cells. VSV was titrated on Vero WHO cells. The limit of detection is 40 PFU/mL. In brief, for both plaque assays, cells were seeded the day before infection (1 ∙ 10^5^ per well in 24-wells plates). Serial dilutions of virus supernatant were added to the cells followed by 2 h incubation at 37 °C prior to placing an overlay of 1% seaplaque agarose (Lonza, Switzerland) in MEM (Gibco). Cells were fixed at 1 (VSV) or 6 days post infection (DENV2). Plaques were visualized with crystal violet (TCS biosciences, UK).

The number of genome-containing particles was determined as previously described^34^ with minor modifications.

### Virus labelling

DENV2 was labelled with DiD (Molecular Probes, USA) as described before^35^. Virus labelling with PKH67 (Sigma, USA) was performed based on Balogh *et al*.^36^. Briefly, 1.5 ∙ 10^8^ GCPs of tartrate-purified DENV2 was diluted in PBS to 100 μL and mixed with 1 μL PKH67 dye in 99 μL Diluent C (Sigma, USA). At 30 seconds post addition, 300 μL of pure FBS was added to stop the labelling. PKH67-labelled virus was used directly after labelling.

### Infection of macrophages

Prior to infection, two wells were trypsinized using 10xTrypsin/EDTA (Gibco) and counted to determine the required amount of virus for each multiplicity-of-infection (MOI). To standardize the opsonisation in ADE experiments, a fixed ratio of antibodies to virus particles was used; per 1 · 10^5^ PFU of DENV2, we added 3 ng of antibody in 75 μL total volume. Thereafter, viruses and antibodies were incubated for 1 h at 37 °C prior to addition to the cells. Cells were washed with warm RPMI and infected at the indicated MOI in 200 μL per well. At 2 hpi, cells were washed to remove extracellular virus and incubation was continued in culture medium (RPMI, 20% FBS, and 10 ng/mL M-CSF). For virus titrations, culture samples were snap-frozen in liquid nitrogen and stored at −80 °C until analysis.

For binding and entry studies, the virus was added to the cells and left to incubate for the designated time periods. At the end, the inoculum was removed, and the cells were lysed in AVL buffer (Qiagen) to determine the total number of particles by qRT-PCR. For the entry studies, cells were gently rocked for 2 min with high-salt-high-pH shaving buffer (1M NaCl, pH 9.5) prior to lysis in AVL buffer.

Flow cytometric of DENV-infected macrophages was performed using 4G2 antibody (Millipore, UK) and donkey anti-mouse IgG-coupled to AF647 (Molecular Probes). Hereto, trypsinized macrophages were fixed with 4% paraformaldehyde in PBS and permeabilized with saponin (Sigma, USA). Flow cytometry was performed on a FACScalibur (BD Biosciences) and analysed using FlowJo 7.6.2 or Kaluza 1.1.

Viral protein translation was determined using the mean fluorescence intensity of infected cells, Hereto, infected cells were gated in Kaluza and the mean fluorescence intensity of AF647 was determined for each sample. Values were expressed as percentage of the maximal fluorescence intensity observed within the donor/experiment. Extracellular virus particles did not contribute to the fluorescence signal.

Macrophage IFNαβR signalling was blocked using the mouse anti-human IFNαβR2 (Millipore) by pre-incubating the cells for 2 h with 0.5 μg/well of antibody. Then, cells were infected and cultured as usual, in the presence of the antibody (1 μg/mL). Infection was scored at 26 hpi and 48 hpi by qRT-PCR. Specificity of IFNαβR blocking was confirmed by quantifying surface expression of CD14 and MHC class I by flow cytometry.

For the interferon-alpha add-on experiments; macrophages were infected as described above. At the designated time points, 1 unit/mL of recombinant interferon α2a (Prospec-Tany, 2.7 · 10^8^ IU/mg) was added to the culture and the concentration was maintained throughout the remainder of the experiment. At 26 hpi, virus particle production was determined by qRT-PCR.

### Fusion assay on primary human macrophages

Isolation and culture of macrophages was initiated as described above. On the second day of culture, the cells were gently dissociated by incubation at 4 °C for 15 minutes followed by gentle pipetting. Subsequently, cells were reseeded at 2 ∙ 10^5^ per quadrant in 500 μL in CELLview dishes (Greiner Bio-One, DE). Subsequent culture and infection was done as above. At 30 min post-infection, extracellular virus was removed by gentle washing the cells with shaving buffer followed by fixation with 4% PFA-PBS. Fusion activity was determined as described before^35^. Briefly, wide-field microscopy analysis was done by taking 15–30 random snapshots using both differential interference contrast and DiD-channels in a Leica Biosystems 6000B instrument with a 635-nm helium-neon laser. Acquired images were analysed with ImageJ using an in house macro^35^ measuring the total fluorescent signal per field of view with the “Particle analyzer” plugin of ImageJ. Fluorescence intensity was normalized relative to MOI 1. The fraction of fusion-positive cells, with at least one bright fluorescent spot, was blindly scored.

### Antiviral bio-assay

The antiviral response in culture supernatants was determined using VSV and Vero WHO cells as described before^25^. Briefly, Vero WHO cells were seeded in 12-wells plates at 2 ∙ 10^5^ per mL per well. After adherence for 8 h, cells were incubated for 12 h with UV-inactivated supernatant, followed by infection with VSV at an MOI of 0.1 in 100 μL. At 1 hpi, inoculum was removed and cells were washed. Incubation was continued in fresh UV-inactivated supernatant. At 6 hpi, medium was collected and VSV titers were determined by plaque assay. Experimental results are reported as percentage of mock-medium. Recombinant human interferon–alpha (Prospec-Tany) served as positive control.

### Microarray

Macrophages were infected at MOI 1, MOI 1-ADE and MOI 1-IgG. Matched infection controls represent conditions resulting in similar fractions of infected cells; i.e. infection at MOI 5 or MOI 2½ (3 and 1 donor at 2 hpi, 2 and 1 donor at 24 hpi, respectively). Cellular RNA was extracted with RNeasy Plus (Qiagen) as per manufacturer’s instructions. RNA was processed *in house* and randomly annealed to Human HT-12 V3 BeadChip array (Illumina, USA), as per manufacturers’ protocol. Data was converted using GeneSpring (Agilent Technologies, USA). Probe values were normalized against the total signal intensity of the sample and subsequently, the fold change of the probes were expressed relative to mock condition of the same donor. For each time point, the donors were pooled and averaged. Probes with an absolute, average fold-change of ≥1.5 relative to the mock were considered differentially regulated. Probes were manually curated to single gene level, independent of the potential isoforms, and Venn diagrams were drawn based on overlaps between infection conditions. All data is been freely available through ArrayExpress ID: E-MTAB-3138.

### DAVID

[Database for Annotation, visualization, and Integrated Discovery]^37^ Pathway analysis was performed to functionally annotate gene groups using standard criteria and an EASE of 0.1. Interferome database^38^ was used to identify an IFN signature in the gene selections, using standard criteria with absolute fold change of ≥2 and limited to the haematopoietic system^38^.

### Statistical analysis

Statistical analysis was performed using Prism 5.00 (Graphpad, USA). A two-sided student’s t-test was used throughout the paper to determine the significance of enhancement after antibody-mediated infection. The antiviral response was analysed using One-way ANOVA with Bonferroni compensation. A p-value of ≤0.05 was considered significant for both tests.

## Results

### Human monoclonal antibodies enhance dengue virus infection of primary human macrophages

Primary macrophages are considered important players in ADE of DENV infection^12^,^39^, yet most of the mechanistic studies conducted so far have been conducted in cell lines^26^,^27^,^40^. Although working with primary cells is more challenging than cell lines given the inherent variability between blood donors and the less pronounced ADE effects in primary cells, we feel that it is important to dissect the fundaments of ADE in cells that are thought to contribute to ADE during natural infection. Primary human macrophages were generated by culturing isolated blood monocytes for 6 days in the presence of 100 ng/mL M-CSF. The resulting cells showed a typical macrophage expression pattern (Fig. A1). In line with previous literature^31^,^41^, primary human macrophages were susceptible to DENV2 infection in an MOI-dependent manner (between MOI 0.2 and MOI 10) (Fig. A2, and Table 1).

**Figure 1:**
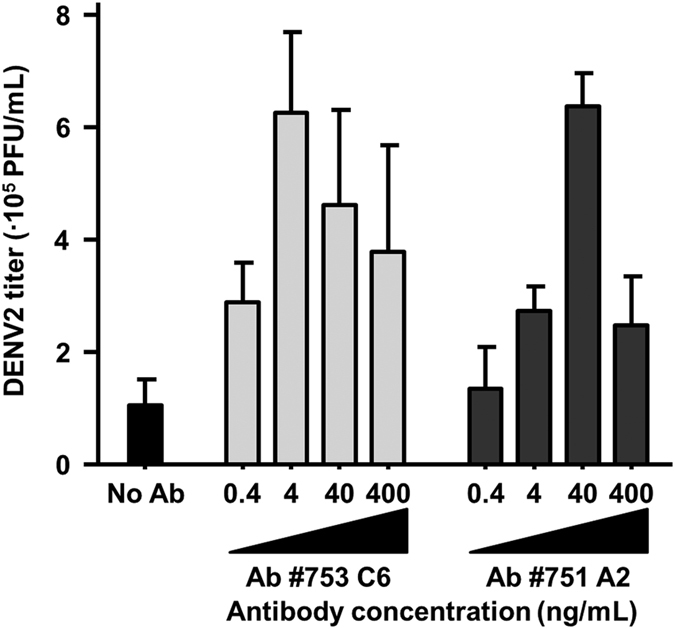
Antibody-dependent enhancement of DENV2 infection of primary human macrophages is dose-dependent. Macrophages were incubated with DENV2, strain 16681 at MOI 1 which had been pre-incubated for 1 h with increasing concentrations of monoclonal human antibody (#753 C6; light grey, or #751 A2; dark grey). At 48 hpi, virus production was determined by plaque assay on BHK-15 cells. Shown is the SEM of duplicates. The figure is representative for two independent experiments. These antibodies are described more in-depth in a previous publication^42^.

To test for ADE, we used human monoclonal antibodies against distinct epitopes of the DENV envelope protein; *e.g.* domains (D) I/DII (#753 C6), and the DII fusion loop (#751 A2). These antibodies were previously shown to be cross-reactive against all four serotypes of DENV^42^. Notably, we found that the power of enhancement was similar between the antibodies (Fig. 1) and independent of the MOI (Fig. A2). Given the overlapping results between the antibodies, we decided to focus on the E DII fusion loop antibody #751 A2 for the remaining experiments. Peak enhancement was observed at an antibody concentration of 40 ng/mL, giving 6.8 ± 1.3-fold enhancement (N = 9) at MOI 1 (Fig. 1). The observed power of enhancement is comparable to other studies using primary human cells^23^,^24^,^25^.

In line with the current hypothesis of ADE, ADE enhanced both the infected cell mass and the burst size (Table 1). The percentage of infection was scored at 26 h post infection (hpi) by flow cytometry. The burst size was calculated by dividing the virus titer as measured with plaque assay by the number of infected cells. In absence of antibodies, 2.0% ± 0.5 of the cells were infected with DENV at MOI 1. Under conditions of MOI 1-ADE, the fraction of infected cells increased to 3.7% ± 0.9 (Table 1). For individual donors, the average enhancement was 2.3 ± 0.6-fold (MOI 1 *versus* MOI 1-ADE, p = 0.023, N = 4 donors). At the same time, the burst size increased 4.2 ± 0.4-fold (N = 4, p = 0.005). The increase in burst size indeed suggests that intrinsic ADE mechanisms are involved. Interestingly though, infection at MOI 10 in the absence of antibodies also increased the burst size (Table 1). Increased burst sizes were already observed at MOI 2½, and infection at MOI 5 closely mimicked MOI 1-ADE in terms of burst size and infected cell mass (Table A1).

To determine whether ADE influenced the specific infectivity of the progeny virus (particles per PFU), we quantified the number of genome-containing particles (qRT-PCR) and the number of infectious particles (plaque assay). In contrast to results from cell lines^26^, we found that neither antibodies nor MOI influenced the particle/PFU ratio of DENV2 in primary human macrophages (Table 1). This suggests that the observed ADE effect occurs prior to assembly, maturation and secretion of progeny virions.

### Enhanced transcription and translation during ADE, yet the replication efficiency is unaffected

**Figure 2:**
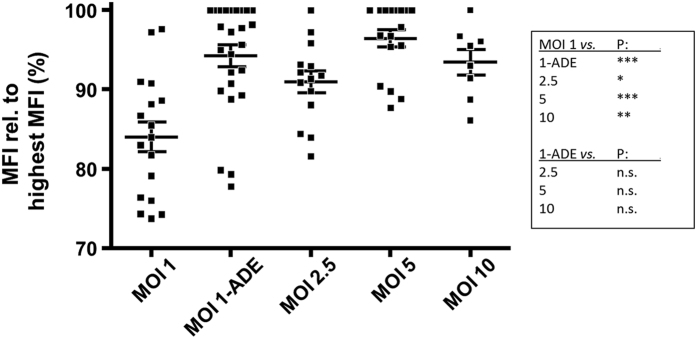
Antibodies and infection at high MOI enhance virus translation. Viral translation was determined per cell on the intracellular pool of envelope protein. Macrophages were infected with DENV2. At 24–26 h, cells were stained with the anti-envelope antibody 4G2 and analysed by flow cytometry. Mean fluorescence intensities were normalized to the sample with the highest intensity of the donor. Up to 11 blood donors were used and six donors were used twice. Statistical analysis was done by One way ANOVA with Bonferroni post-test correction; *(p ≤ 0.05), **(p ≤ 0.01) ***(p ≤ 0.0001), n.s.: non-significant.

We next studied the effect of antibodies on protein translation and viral genome replication using flow cytometry and qRT-PCR, respectively. Protein translation was determined on a per-cell-basis by measuring the mean fluorescence intensity (MFI) of the envelope proteins within the cell. Figure 2 shows that the MFI is clearly enhanced under conditions of ADE at 24 hpi (N = 11, p ≤ 0.0001), suggesting that ADE may enhance viral protein translation. Higher concentrations of virus (MOI 2½, MOI 5, and MOI 10), in line with the increased burst size, also resulted in higher E protein content per cell yet protein translation did not differ among the higher MOI’s (Fig. 2).

Next, the replication efficiency of the virus was determined at 24 hpi by measuring the intracellular ratio of positive-sense and negative-sense RNA. ADE leads to 10-fold higher numbers of both negative- (9.8 ± 4.4, N = 3) and positive-sense (9.8 ± 3.5, N = 4) RNA. Yet, the ratio of positive *versus* negative RNA was comparable between MOI 1 and MOI 1-ADE (4.9 ± 1.0 *versus* 4.8 ± 2.7, respectively (N = 4)), suggesting that antibody-mediated infection does not influence the replication efficiency of DENV. Contrary to this, infection at MOI 5 showed a ratio of 2.5 ± 1.5 (N = 4), indicating that the replication efficiency is negatively affected under conditions of higher MOI’s.

Thus, the increased burst size during ADE is associated with enhanced transcription and translation while the replication efficiency is similar to MOI 1. In absence of antibodies, similar infectivity, burst size and translation can be attained using higher MOIs yet at the cost of the replication efficiency.

### Antibodies enhance fusion while maintaining the same cellular dose of virus particles

It is assumed that the higher number of genome copies and increased protein translation is a consequence of higher virus cell binding and/or increased uptake of particles into cells. Therefore, we quantified virus binding and cell entry using qRT-PCR. To measure viral entry, extracellular virions were removed by washing the cells with a high-salt-high-pH buffer^43^.

**Figure 3:**
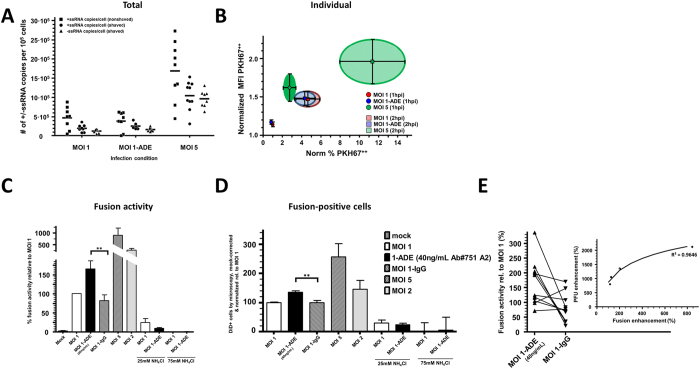
Antibodies do not alter the efficiency of dengue virus to bind or enter into primary human macrophages, whilst promoting fusion within primary macrophages. (**A**) DENV2 binding and uptake in primary macrophages was determined at 1 hpi by qRT-PCR using template-specific primers in combination with RNAse A treatment. Extracellular virus was removed by shaving the cells with a high-salt-high-pH buffer for 2 min. Squares show the total number of virus particles that had bound or entered cells. Circles depict entered viral genomes, while triangles show negative-sense RNA genomes. Shown are 4 donors with each condition in duplicate. (**B**) Macrophages were infected with PKH67-labelled DENV at MOI 1 (red), MOI 1-ADE (blue) or MOI 5 (green). At 1 h (filled) or 2 h (striped) of incubation, the cells were shaved and fixed prior to analysis by flow cytometry. Cell entry was normalized to MOI 1, and the mean fluorescence intensity (MFI) was normalized to the negative control. Shown are the SEM of 5 donors. At MOI 1, 13 ± 3% of the cells were positive for PKH67-labelled DENV entry. (**B–E**) The fusion activity of DENV2 within primary human macrophages was determined at 30 min by microscopy using the self-quenching fluorophore DiD. Pictures were taken randomly and analysed for fusion activity (**C**), and the fraction of fusion-positive cells (**D**). All values were normalized to MOI 1 of the same donor (**C**) or infection in absence of antibodies (**D,E**). At MOI 1, the average number of fusion-positive cells was 17 ± 2.2%. (**E**) An overview of connected values of fusion activity when primary human macrophages are infected in the presence of enhancing antibodies (ADE), and when infected in the presence of an isotype (IgG). The inset shows the correlation between enhancement of fusion activity at 30 min post infection and the subsequent enhancement in virus production at 26 hpi. All values are given as percentage of the results obtained at MOI 1. Shown are the normalized SEM of up to 12 donors (**A–C**) and 5 experiments (**D,E**). Statistical analysis was done by 2-tailed t-test; *(P ≤ 0.05), **(P ≤ 0.01), ***(P ≤ 0.0005).

Overall, virus cell binding (squares) and entry (circles) was not enhanced under conditions of ADE (N = 4 donors) (Fig. 3A). We were surprised by these findings, yet we were not able to use qRT-PCR at later time points due to the initiation of replication as visualized by the negative sense RNA (triangles, Fig. 3A). Therefore, we next used flow cytometry to assess the binding and entry dynamics of DENV2 in individual cells using PKH67-labelled DENV particles. PKH67 is a fusion-independent lipophilic dye that intercalates into the viral membranes^36^. This approach allows us to measure viral uptake and reveals the population of cells that are positive for PKH67. DENV2 was successfully labelled with PKH67 (Fig. A3).

Figure 3B shows the extent of DENV2 uptake against the cell population positive for uptake at 1 hpi (filled shape) and 2 hpi (striped shape). The extent of virus uptake per cell as well as the fraction of positive cells increased over time. Comparable results were obtained for MOI 1 (blue) and MOI 1-ADE (red). Yet, at MOI 5 (green) both the fraction of PKH67-positive cells and the extent of viral uptake per cell were higher than MOI 1/1-ADE at both time points (Fig. 3B). These results are in line with the qRT-PCR data (Fig. 3A), and confirm that ADE does not enhance the binding- or entry-efficiency of DENV2 in primary macrophages.

**Figure 4:**
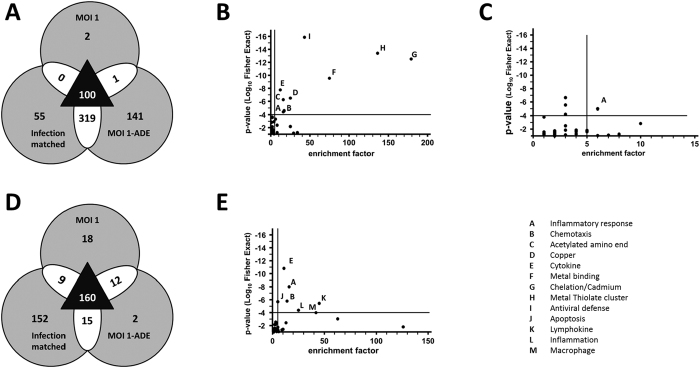
Dengue virus infection of macrophages induces an antiviral response, which depends on the viral load. (**A,D**) Primary human macrophages were infected with DENV2 at MOI 1, MOI 1-ADE, and matched infection (MOI 5 or MOI 2½). Total RNA was isolated at 24 h (**A**) and 2 h (**D**). Gene expression was investigated by microarray. Genes whose average expression had an absolute fold change of at least 1.5-fold over the mock were selected. The Venn diagram shows how these genes are connected with the various infection conditions. (A) is based on 3 donors and (D) on 4 donors. (**B,C,E**) Gene ontology of DAVID pathway analysis was used to annotated genes at 24 h (**B,C**) and at 2 h (**E**). (**B**) shows the ontological analysis of the genes that were shared among the three conditions (see A, triangle, 100 genes).(**C**) shows the analysis of the ADE-effect (see A, 460 genes total; 318 + 142). (**E**) shows the ontological analysis of the genes that were shared among the three conditions at 2 h (see D, triangle, 160 genes). The x-axis shows the enrichment of the term within our selection relative to the DAVID database. The y-axis shows the significance calculated with 1-tailed Fisher Exact statistical analysis. Gene ontology terms with at least 5-fold enrichment and a p-value of ≤1 ∙ 10^−4^ are considered relevant.

Hence, we hypothesized that antibodies enhance a step downstream of entry and prior to replication. For example, antibodies may direct the virus to an organelle and/or cellular location that is more beneficial for membrane fusion and infection. Alternatively, antibodies may enhance the intrinsic fusion capacity of the virus. To assess if antibody-mediated DENV entry increases the fusion potential of the virus, we employed a microscopic fusion assay involving DiD-labelled DENV. Fusion is observed as a sudden increase in fluorescence intensity due to dilution of the probe in the target membrane^34^,^35^,^44^. Figure 3C shows that this assay is specific and robust since the extent of fusion was MOI-dependent and inhibited by ammonium chloride (Fig. 3C, ref. 34). Importantly, membrane fusion activity of DENV under conditions of ADE was enhanced with 65% (Fig. 4A). Furthermore, the number of fusion-positive macrophages increased with 40% (Fig. 3D). The extent to which fusion is enhanced is variable between donors and ranged from 115% to 336% (Fig. 3E inset). Importantly, when the extent of fusion enhancement is plotted against those for the PFU production, the results are correlating with each other, indicating a causal relationship between the two parameters (Fig. 3E).

Recently, we performed similar experiments in the macrophage-like cell line P338D1 (Ayala *et al*., accompanying manuscript). In contrast to our results for primary human macrophages, we did find enhanced binding/entry of DENV in P338D1 cells under conditions of ADE and subsequent higher fusion activity (3.85 ± 0.5 and 3.52 ± 0.64 fold change, respectively). This suggests that the qRT-PCR is able to detect differences in binding and entry where they occur, but also that the mechanism of ADE is cell-type-specific.

We next attempted to assess if antibodies itself influence the fusion potential of the virus using a cell-free liposomal system. However, despite our experience in virus-liposome fusion studies^45^,^46^,^47^, we were not able to detect fusion of DENV with negatively-charged liposomes (data not shown). Thus we were not able to examine whether antibodies intrinsically promote membrane fusion of DENV.

To summarize, ADE in both models led to more fusion-positive cells and more fusion activity per cell (*i.e.* higher genome delivery). The relatively small increase in fusion activity probably initiates a cascade leading to higher infection rates and burst sizes. In primary human macrophages, the efficiency of infection and the burst size is however not solely dependent on membrane fusion activity since MOI 5 and ADE had a similar infected cell mass and burst size, while at MOI 5 a five-fold higher fusion activity was seen compared to ADE. This suggests that the infection process is negatively influenced at MOI 5 compared to MOI 1-ADE. Therefore, we next wished to better understand what happens within the cell to-be-infected.

### Gene profiles of DENV-infected macrophages discriminate between high and low infection

To identify the cellular responses during DENV infection, we profiled the gene expression patterns at 2 and 24 hpi. Macrophages were infected at MOI 1, and MOI 1-ADE to study the biological process of ADE. Also, infection-matched controls, with a similar fraction of infected cells (MOI 2½ or MOI 5, depending on the donor), were included to better understand the increased burst size observed at high MOI. As controls, we included non-infected cells and infections at MOI 1 in the presence of non-relevant antibodies (1-IgG). All donors and conditions were normalized to the mock, and genes with a fold-change of ≥1.5 of the mock were selected. Venn diagrams were drawn to visualize the overlaps between various conditions of infection.

First, we studied the biological process of ADE at 24 hpi.The Venn diagram for this time point shows that ADE induces a strong alteration in the transcriptional profile of infected human macrophages; 101 genes were shared between MOI 1 and MOI 1-ADE, while 460 genes were uniquely for ADE. Contrary to this, only 2 genes were unique to infection at MOI 1 without antibodies.

**Figure 5:**
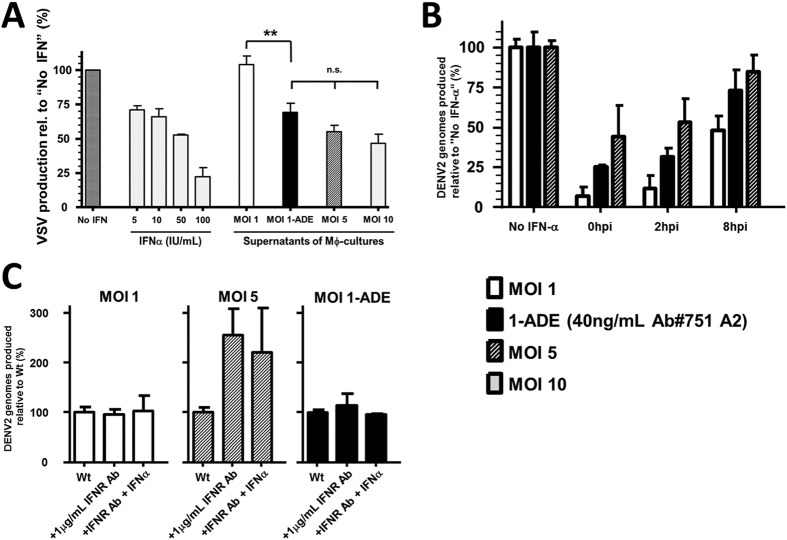
Early type-I-interferon (IFN)-mediated responses significantly determine the outcome of infection. (**A**) Primary macrophages were infected with DENV2 at MOI 1, MOI 1-ADE, MOI 5, and MOI 10. Culture supernatants were harvested at 24 h and UV-inactivated. Inactivated supernatants were used to pre-activate Vero cells prior to infection with VSV at MOI 0.1. Shown is the SEM of 3 donors, with infection performed in duplicate and duplicates of this assay. Statistical analysis was done with One way ANOVA with post-test Bonferroni compensation; **(p ≤ 0.01) and n.s.; non-significant. (**B**) IFNα protects primary human macrophages against DENV2 infection when applied during the early (0–2 hpi) time points, and not during late (8 hpi) time points. Macrophages were infected with DENV2 at MOI 1, MOI 1-ADE and MOI 5. At the designated time points, 1 IU of recombinant human IFNα2a was added to the culture. At 26 hpi, the viral titre in the supernatant was determined by qRT-PCR and normalized relative to the unperturbed condition (No IFNα). Shown is the SEM of 4–5 donors, each condition in duplicate. (**C**) Type I IFN signalling was blocked by pre-incubating macrophages for 2 h with an antibody against the IFNαβR. After incubation, cells were infected at MOI 1, MOI 1-ADE and MOI 5. Supernatants were sampled at 26 hpi and virus production was determined by qRT-PCR. Addition of 10 units of IFNα at 0 hpi served as a control for IFNαβR-blocking. Shown is the normalized SEM of 2 donors, each condition in duplicate.

Yet, a comparison of MOI 1-ADE with the infection-matched control shows that a large majority of the ADE-genes are associated with high infection (319 genes, Fig. 4A). Thus, the transcriptional response upon ADE is likely induced by the larger infected cell mass and/or higher viral load in the supernatant (2-fold and 8-fold higher than MOI 1, respectively). DAVID pathway analysis^37^ was used to functionally annotate the gene patterns (Fig. 5B,C). We were interested in two sets of genes: the shared genes (100 genes, Fig. 4A) and the ADE-associated genes (141 + 319 genes, Fig. 4A). Gene ontology identified the term “antiviral defense” as the most significant term for the shared genes (Fig. 4B). For the ADE-associated genes, the sole significant term was “inflammatory responses” (Fig. 4C), hence antibodies did not induce a long-lasting immune-suppressive state in our macrophage cultures.

Hence, we focused on the transcriptional profiles shortly after infection. At 2 hpi, both MOI 1 and MOI 1-ADE had very similar transcriptional profiles (Fig. 4D). Contrary to this, MOI 5 showed a much stronger shift in its transcriptional profile. The lack of difference between MOI 1 and MOI 1-ADE at 2 hpi is in contrast with the 24 hpi time point (Fig. 4A,D, respectively), suggesting that antibodies do not induce specific transcriptional profiles. A comparison of the profiles induced by MOI 1, MOI 1-IgG and MOI 1-ADE indeed revealed that infection in the presence of DENV antibodies does not induce specific pathways (Fig. A4). At 2 hpi, 160 of the genes were shared between the three conditions (triangle, Fig. 4D). Gene ontology analysis of these 160 shared genes identified an inflammatory response (Fig. 4E). No specific suppressive pathway was identified in the MOI 1-ADE cluster (Fig. 4E). Thus, at both time points of infection, antibody-mediated infection did not activate immune-suppressive pathways. Rather, we found high induction of the cytokines IFNβ, TNFα, IL1β, IL6 and little to no IL10 (Fig. A4B). Moreover, the fraction of interferon (IFN)-regulated genes^38^ is quite substantial (Table 2), suggesting that there was an antiviral state in our cultures despite the antibody-dependent infection mechanism.

### Antibodies enhance fusion while avoiding additional antiviral responses

Microarrays provide a snap-shot of the expression profile at the mRNA level. To confirm the antiviral state, we next analysed the antiviral activity of the macrophage supernatants described above (Fig. 5A). As different cytokine profiles can confer the same protection^48^, we used a viral bio-assay^25^ combining the IFN-sensitive VSV^49^ with the IFN-deficient yet -sensitive Vero WHO cell line^50^. At 24 hpi, no antiviral activity towards VSV was observed in the supernatants of macrophages infected at MOI 1 of DENV without antibodies. Yet, following infection at MOI 1-ADE, MOI 5 and MOI 10, antiviral activity was seen (Fig. 5A). This indicates that antibodies *per se* do not induce a virus-tolerant environment by 24 hpi. Moreover, antiviral responses appear to increase with the percentage of DENV2-infected macrophages (Fig. 5A, Table 1 and A1), indicating a dose-dependent response.

At 24 hpi, MOI 5 had a comparable number of infected cells, antiviral activity and burst size as MOI 1-ADE. Yet, MOI 5 had a 5-fold higher fusion activity and a 2-fold lower replication efficiency. Moreover, more genes are potentially IFN-regulated (Table 2). Earlier studies in human cell lines showed that the sensitivity of DENV to IFN is most pronounced during early stages of the viral life cycle, and over time the virus becomes resistant to the antiviral activity of IFN^43^. Hence, we hypothesized that a high MOI of DENV2 induces a stronger, type-I-IFN-mediated response early in infection thereby lowering the replication efficiency. To confirm our hypothesis, we first attempted to quantify the concentrations of IFNα and IFNβ at 2 hpi in the macrophage supernatants from the screen by ELISA. The IFNβ concentration was below the limit of detection for all conditions, and the IFNα concentration fluctuated between the donors and conditions likely due to the very low concentrations measured (Table A2).

To evaluate if DENV also becomes resistant to IFN in primary macrophages, we added exogenous IFN to cells at different time points post-infection and assessed virus production. Indeed, and in line with earlier results, the antiviral activity of IFNα is most pronounced when added early in infection (Fig. 5B). At late time points, the effect of exogenous IFN diminishes indicating that once infection is established the antiviral effect of IFN is limited. Importantly, the effect of exogenous IFN was distinct between the conditions tested with the lowest effect at MOI 5 and the strongest effect at MOI 1. The higher resistance at MOI 5 is indicative for an endogenous IFN response early in infection, since the added IFN had a lower contribution to the antiviral state already present in the culture. Interestingly, Fig. 5B also shows that the addition of just one unit of IFNα to the cells at the time of infection significantly reduces ADE (4-fold reduction in virus production), indicating that ADE is sensitive to IFN.

If, at MOI 5, the early endogenous IFN response reduces virus particle production, then blocking the IFNαβR prior to infection should lead to an increase in virus production. Indeed, specific blocking type-I-IFN receptor signalling significantly enhanced virus production at MOI 5 (Fig. 5C and A5). Interestingly, and in contrast, infection at MOI 1 or MOI 1-ADE was not enhanced by the IFNαβR antibody, confirming that MOI 1 and MOI 1-ADE do not trigger IFNαβR-signalling during the early stages of infection. This suggests that the efficiency of DENV2 infection in primary human macrophages is determined by the balance between fusion activity and antiviral responses early in infection. Once DENV infection and replication is established, the antiviral response no longer determines virus production (Fig. 5A(+24 hpi) and 5B(+8 hpi)), likely due to the viral proteins^51^.

## Discussion

Antibody-mediated cell entry of DENV is known to increase the infected cell mass and virus particle production, but little is known about the underlying mechanisms. Our results show that, in primary macrophages, antibodies enhance DENV infection by promoting fusion (Fig. 3C,E), while no altered uptake of virus particles is seen (Fig. 3A,B). The higher fusion potential triggers a cascade of events leading to enhanced infection, replication, translation, and burst size. Furthermore, the presence of enhancing concentrations of antibodies did not trigger pro- or anti-viral programs early in infection (Fig. 4E). At high MOI, however, increased binding, uptake, fusion and an increased antiviral response is seen early in infection (Figs 3A–D and 5C).

Our results show that fusion is the first step within primary macrophages where antibodies have a positive influence on the life cycle of DENV. Indeed, not only the total extent of virus cell binding but also the overall distribution of virus particles among cells was comparable in the absence and in the presence of antibodies (Fig. 3A,B). The increase in membrane fusion activity does, however, not directly translate into the observed increase in the burst size. Yet, it is interesting to note that the extent of membrane fusion activity correlates with virus production (Fig. 3E inset). This is suggestive for a causal relationship between fusion activity and virus production in primary macrophages. Further research is required to investigate the downstream effects of ADE more in-depth. Important steps to elucidate herein are for example the effect on nucleocapsid delivery, nucleocapsid uncoating and subsequent initiation of protein translation.

**Figure 6:**
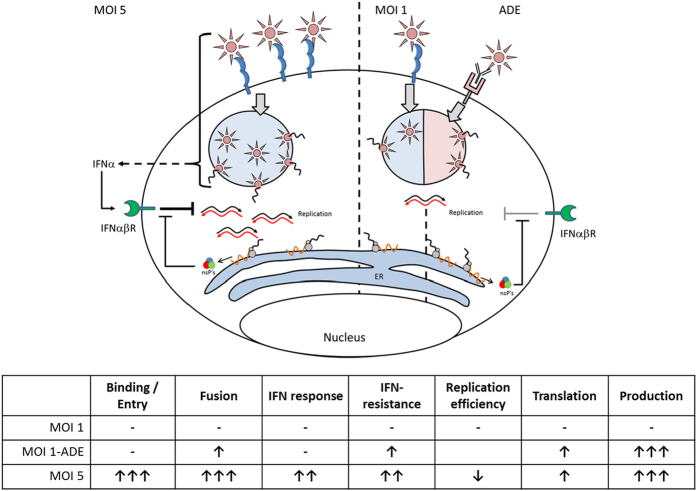
Molecular mechanisms involved in antibody-dependent enhancement of dengue virus infection in primary human macrophages. Antibody-dependent infection does not enhance binding or entry of the virus to the cells. Yet, the membrane fusion potential within the endosomes of the macrophage is increased. Thanks to the unaltered characteristics of binding and entry, ADE does not trigger endogenous interferon-responses which thus allows the virus to replicate freely during the early stages of infection. ADE can be mimicked in terms of the number of infected cells and burst size by infection at high MOI in absence of antibodies. Yet high MOI leads to more binding, entry, fusion, and as a consequence induction of an IFN response. The presence of an early IFN response significantly reduces virus replication and production. ADE is thus based on higher fusion but due to the absence of an early IFN response, it remains unnoticed by the cell allowing virus replication to higher titres.

We propose that the “normal” binding and entry characteristics of ADE is key to the success of ADE in primary macrophages as this avoids extensive activation of antiviral signalling early in infection (Fig. 5B). Indeed, blocking IFNαβR-signalling rescued DENV production at MOI 5 but had no effect at MOI 1 or 1-ADE (Fig. 5C). Contrary to MOI 5, ADE displays enhanced fusion activity while maintaining low antiviral responses (Fig. 5C). Collectively, our results suggest that ADE of DENV infection in primary macrophages involves a novel mechanism that is tightly balanced between the extents of binding, entry, fusion and the antiviral responses early in infection (Fig. 6).

In contrast to our results in primary macrophages, we did observe enhanced binding of DENV-immune-complexes in macrophage-like P338D1 cells (Ayala *et al*., accompanying manuscript). In P338D1 cells, we noted 3.85-fold higher binding/uptake of DENV into the cells and 3.52-fold higher fusion activity, which suggests that ADE in P338D1 cells is facilitated by an increased binding efficiency of the virus-immune-complexes to the cell. These results suggests that alternative, cell-type-specific, mechanisms exists to facilitate ADE. An alternative explanation for our findings in primary macrophages is that virus particles are non-randomly distributed and cells that support ADE are more permissive than other cells. While this notion deserves further research, it seems unlikely as the overall distribution of virus particles was comparable in the presence and in the absence of antibodies (Fig. 3B).

ADE could be mimicked in terms of infected cell mass and burst size by infection at high MOI. Furthermore, strong overlapping transcriptional profiles and antiviral responses were observed late in infection. This suggests that antibodies do not trigger specific intrinsic pathways. Indeed, no antibody-specific anti-inflammatory program was found in primary human macrophages (Fig. 5D and A4). This in agreement with a recent whole blood transcriptome analysis^52^ and in contrast with studies using PBMCs^19^ or cell lines^53^. Furthermore, other studies described a “muted” response^54^,^55^, but argued that the actual response could have waned by the time of sampling^54^,^56^. Therefore, the antibody-dependent immune suppression could be specific for the cell type(s) investigated.

In monocytes, the anti-inflammatory cytokine IL10 is considered to be one of the driving forces of intrinsic ADE^29^,^53^. In macrophages, however, we and others found low induction of IL10 after ADE (Fig. 5F, refs 25,57), and only at late time points for conditions with high infection and viral load (Fig. 5F, ref. 58). Hence, IL10 does not directly influence ADE of DENV infection in macrophages. In line with this, IL10-primed macrophages are highly susceptible to DENV infection but do not produce progeny virus^59^. This manuscript focused only on primary human macrophages and future research will have to elucidate the role of IL10 in DENV-ADE in other cell types. Based on our results, we propose that the enhanced burst size, as observed in human macrophages, is dependent on the effective MOI (fusion extent) and is not antibody-specific.

Dengue haemorrhagic fever is characterized by vascular leakage, which on its turn has been linked to the high inflammatory response in patients (reviewed in: ref. 60) and the action of the DENV NS1^61^,^62^. Here we show that antibody-dependent DENV infection of primary human macrophages results in 7-fold higher virus titres, and subsequently triggers strong inflammatory responses (Figs 5C,F and 6A,^25^). The link between viral burden and vascular permeability has been described before^24^,^52^,^61^,^62^ and our data further strengthens the notion that dengue haemorrhagic fever might be alleviated by reducing the viral load. Indeed, we show that application of IFNα suppressed virus production by human macrophages, even under conditions of ADE.

An important remaining question is how the antibodies enhanced the fusion efficiency of the internalized particles. There are two options; (i) antibodies directly enhance the fusion potential of the virus, or (ii) the phagosomal environment of primary macrophages is more favourable for fusion. Although we cannot disprove the first hypothesis, it seems unlikely as no epitope-specific effects were seen in ADE (data not shown). Antibody-mediated cell entry is facilitated through interaction of the antibody with the Fc receptor. Due to this interaction, DENV-immune-complexes are internalized via phagocytosis (Ayala *et al*., accompanying manuscript). DENV fusion is critically dependent on pH and negatively charged lipids^34^,^63^. These factors are differentially regulated and distributed between distinct organelles^63^,^64^, and may explain why enhanced fusion is seen in primary macrophages.

In conclusion; antibodies enhance DENV infection of human macrophages by promoting fusion of the virus particles within endosomes. Our work suggests that the higher effective MOI (fusion, translation, replication) causes the enhanced burst size, not the interaction of antibodies with the FcR. We show that even modest enhancement of fusion activity initiates a cascade with increasingly aggravating impacts. Importantly, DENV2-ADE does not suppress antiviral responses, but rather avoids induction hereof. We show that DENV2-ADE can be significantly suppressed by addition of IFNα to primary human macrophages. These IFN-experiments and our microarray show that the dreaded induction of immunosuppression apparently is not involved in DENV-ADE in macrophages. Therefore, the presented work offers new perspectives to treat dengue virus, with fusion and antiviral responses as the key steps between high and low infection in primary human macrophages.

## Additional Information

**How to cite this article**: Flipse, J. *et al*. Antibody-Dependent Enhancement of Dengue Virus Infection in Primary Human Macrophages; Balancing Higher Fusion against Antiviral Responses. *Sci. Rep.* **6**, 29201; doi: 10.1038/srep29201 (2016).

## Supplementary Material

Supplementary Information

## Figures and Tables

**Table 1:** Antibodies enhance both infection and burst size, but not the specific infectivity of progeny virions.

MOI	Infected cells (%)	Burst size (PFU/cell)	Particles/PFU
1	2.0 ± 0.5	2.8 ± 1.0	255 ± 34
1-ADE	3.7 ± 0.9	8.9 ± 2.4	212 ± 19
10	11.3 ± 2.3	17.2 ± 4.7	268 ± 24

Primary human macrophages were infected at MOI 1 or 10, and MOI 1-ADE. For MOI 1-ADE DENV was pre-incubated for 1 h with 40 ng/mL of antibody #751 A2. The number of infected cells was determined by flow cytometry at 26 hpi. Concurrently, the virus titer was determined by both qRT-PCR (physical particles) and plaque assay (infectious particles). The burst size was calculated by dividing the virus titer by the number of infected cells and the specific infectivity resembles the number of physical particles divided by the number of infectious particles. All values are SEM of duplicates of 4 donors.

**Table 2:** High viral load stimulates IFN-regulated genes early in infection.

	Shared	MOI 5 unique	Shared MOI 5 & 1-ADE
2 hpi	30.9% (160 genes)	25.7% (152 genes)	xxx
24 hpi	79.8% (100 genes)	xxx	49.2% (319 genes)

Genes were selected from the Venn diagrams in Fig. 4A (24 h) and 4E (2 h). Selected genes were analysed by mining the Interferome database^38^ for interferon-regulated genes in haematopoietic cells which are reported as the percentage of the selected genes.

## References

[b1] Hawkes R. A. Enhancement of the Infectivity of Arboviruses by Specific Antisera Produced in Domestic Fowls. Aust. J. Exp. Biol. Med. Sci. 42, 465–482 (1964). 14202187 10.1038/icb.1964.44

[b2] Halstead S. B. *In vivo* enhancement of dengue virus infection in rhesus monkeys by passively transferred antibody. J. Infect. Dis. 140, 527–533 (1979). 117061 10.1093/infdis/140.4.527

[b3] Tamura M., Webster R. G. & Ennis F. A. Subtype cross-reactive, infection-enhancing antibody responses to influenza A viruses. J. Virol. 68, 3499–3504 (1994). 8189489 10.1128/jvi.68.6.3499-3504.1994 PMC236853

[b4] Sauter P. & Hober D. Mechanisms and results of the antibody-dependent enhancement of viral infections and role in the pathogenesis of coxsackievirus B-induced diseases. Microbes Infect. 11, 443–451 (2009). 19399964 10.1016/j.micinf.2009.01.005

[b5] Krilov L. R., Anderson L. J., Marcoux L., Bonagura V. R. & Wedgwood J. F. Antibody-mediated enhancement of respiratory syncytial virus infection in two monocyte/macrophage cell lines. J. Infect. Dis. 160, 777–782 (1989). 2809253 10.1093/infdis/160.5.777

[b6] Takada A., Feldmann H., Ksiazek T. G. & Kawaoka Y. Antibody-dependent enhancement of Ebola virus infection. J. Virol. 77, 7539–7544 (2003). 12805454 10.1128/JVI.77.13.7539-7544.2003 PMC164833

[b7] Willey S. . Extensive complement-dependent enhancement of HIV-1 by autologous non-neutralising antibodies at early stages of infection. Retrovirology 8, 16-4690-8-16 (2011). 10.1186/1742-4690-8-16 PMC3065417 21401915

[b8] Suhrbier A. & La Linn M. Suppression of antiviral responses by antibody-dependent enhancement of macrophage infection. Trends Immunol. 24, 165–168 (2003). 12697441 10.1016/s1471-4906(03)00065-6

[b9] Guzman M. G., Alvarez M. & Halstead S. B. Secondary infection as a risk factor for dengue hemorrhagic fever/dengue shock syndrome: an historical perspective and role of antibody-dependent enhancement of infection. Arch. Virol. 158, 1445–1459 (2013). 23471635 10.1007/s00705-013-1645-3

[b10] Kliks S. C., Nimmanitya S., Nisalak A. & Burke D. S. Evidence that maternal dengue antibodies are important in the development of dengue hemorrhagic fever in infants. Am. J. Trop. Med. Hyg. 38, 411–419 (1988). 3354774 10.4269/ajtmh.1988.38.411

[b11] Bhatt S. . The global distribution and burden of dengue. Nature 496, 504–507 (2013). 23563266 10.1038/nature12060 PMC3651993

[b12] Halstead S. B. & O’Rourke E. J. Dengue viruses and mononuclear phagocytes. I. Infection enhancement by non-neutralizing antibody. J. Exp. Med. 146, 201–217 (1977). 406347 10.1084/jem.146.1.201 PMC2180729

[b13] Burke D. S., Nisalak A., Johnson D. E. & Scott R. M. A prospective study of dengue infections in Bangkok. Am. J. Trop. Med. Hyg. 38, 172–180 (1988). 3341519 10.4269/ajtmh.1988.38.172

[b14] Buchy P. . Secondary dengue virus type 4 infections in Vietnam. Southeast Asian J. Trop. Med. Public Health 36, 178–185 (2005). 15906664

[b15] Anderson K. B. . A shorter time interval between first and second dengue infections is associated with protection from clinical illness in a school-based cohort in Thailand. J. Infect. Dis. 209, 360–368 (2014). 23964110 10.1093/infdis/jit436 PMC3883164

[b16] Montoya M. . Symptomatic Versus Inapparent Outcome in Repeat Dengue Virus Infections Is Influenced by the Time Interval between Infections and Study Year. PLoS Negl Trop. Dis. 7, e2357 (2013). 23951377 10.1371/journal.pntd.0002357 PMC3738476

[b17] Endy T. P. . Relationship of preexisting dengue virus (DV) neutralizing antibody levels to viremia and severity of disease in a prospective cohort study of DV infection in Thailand. J. Infect. Dis. 189, 990–1000 (2004). 14999601 10.1086/382280

[b18] Libraty D. H. . Differing influences of virus burden and immune activation on disease severity in secondary dengue-3 virus infections. J. Infect. Dis. 185, 1213–1221 (2002). 12001037 10.1086/340365

[b19] Ubol S. . Differences in global gene expression in peripheral blood mononuclear cells indicate a significant role of the innate responses in progression of dengue fever but not dengue hemorrhagic fever. J. Infect. Dis. 197, 1459–1467 (2008). 18444802 10.1086/587699

[b20] Vaughn D. W. . Dengue viremia titer, antibody response pattern, and virus serotype correlate with disease severity. J. Infect. Dis. 181, 2–9 (2000). 10608744 10.1086/315215

[b21] Moi M. L., Takasaki T., Saijo M. & Kurane I. Dengue virus infection-enhancing activity of undiluted sera obtained from patients with secondary dengue virus infection. Trans. R. Soc. Trop. Med. Hyg. 107, 51–58 (2013). 23296697 10.1093/trstmh/trs007

[b22] Flipse J., Wilschut J. & Smit J. M. Molecular mechanisms involved in antibody-dependent enhancement of dengue virus infection in humans. Traffic 14, 25–35 (2013). 22998156 10.1111/tra.12012

[b23] Blackley S. . Primary human splenic macrophages, but not T or B cells, are the principal target cells for dengue virus infection *in vitro*. J. Virol. 81, 13325–13334 (2007). 17928355 10.1128/JVI.01568-07 PMC2168870

[b24] Boonnak K., Dambach K. M., Donofrio G. C., Tassaneetrithep B. & Marovich M. A. Cell type specificity and host genetic polymorphisms influence antibody-dependent enhancement of dengue virus infection. J. Virol. 85, 1671–1683 (2011). 21123382 10.1128/JVI.00220-10 PMC3028884

[b25] Kou Z. . Human antibodies against dengue enhance dengue viral infectivity without suppressing type I interferon secretion in primary human monocytes. Virology 410, 240–247 (2011). 21131015 10.1016/j.virol.2010.11.007

[b26] Quinn M., Kou Z., Martinez-Sobrido L., Schlesinger J. J. & Jin X. Increased virus uptake alone is insufficient to account for viral burst size increase during antibody-dependent enhancement of dengue viral infection. J. Immunol. Tech. Infect. Dis. 2, 3 (2013).

[b27] Modhiran N., Kalayanarooj S. & Ubol S. Subversion of innate defenses by the interplay between DENV and pre-existing enhancing antibodies: TLRs signaling collapse. PLoS Negl Trop. Dis. 4, e924 (2010). 21200427 10.1371/journal.pntd.0000924 PMC3006139

[b28] Ubol S. & Halstead S. B. How innate immune mechanisms contribute to antibody-enhanced viral infections. Clin. Vaccine Immunol. 17, 1829–1835 (2010). 20876821 10.1128/CVI.00316-10 PMC3008185

[b29] Ubol S., Phuklia W., Kalayanarooj S. & Modhiran N. Mechanisms of immune evasion induced by a complex of dengue virus and preexisting enhancing antibodies. J. Infect. Dis. 201, 923–935 (2010). 20158392 10.1086/651018

[b30] Dejnirattisai W. . A complex interplay among virus, dendritic cells, T cells, and cytokines in dengue virus infections. J. Immunol. 181, 5865–5874 (2008). 18941175 10.4049/jimmunol.181.9.5865

[b31] Miller J. L. . The mannose receptor mediates dengue virus infection of macrophages. PLoS Pathog. 4, e17 (2008). 18266465 10.1371/journal.ppat.0040017 PMC2233670

[b32] Jessie K., Fong M. Y., Devi S., Lam S. K. & Wong K. T. Localization of dengue virus in naturally infected human tissues, by immunohistochemistry and *in situ* hybridization. J. Infect. Dis. 189, 1411–1418 (2004). 15073678 10.1086/383043

[b33] Richter M. K. . Immature dengue virus is infectious in human immature dendritic cells via interaction with the receptor molecule DC-SIGN. PLoS One 9, e98785 (2014). 24886790 10.1371/journal.pone.0098785 PMC4041791

[b34] van der Schaar H. M. . Characterization of the early events in dengue virus cell entry by biochemical assays and single-virus tracking. J. Virol. 81, 12019–12028 (2007). 17728239 10.1128/JVI.00300-07 PMC2168764

[b35] Ayala-Nunez N. V., Wilschut J. & Smit J. M. Monitoring virus entry into living cells using DiD-labeled dengue virus particles. Methods 55, 137–143 (2011). 21855634 10.1016/j.ymeth.2011.07.009

[b36] Balogh A. . A simple fluorescent labeling technique to study virus adsorption in Newcastle disease virus infected cells. Enzyme Microb. Technol. 49, 255–259 (2011). 22112508 10.1016/j.enzmictec.2011.06.005

[b37] Huang da W., Sherman B. T. & Lempicki R. A. Systematic and integrative analysis of large gene lists using DAVID bioinformatics resources. Nat. Protoc. 4, 44–57 (2009). 19131956 10.1038/nprot.2008.211

[b38] Rusinova I. . Interferome v2.0: an updated database of annotated interferon-regulated genes. Nucleic Acids Res. 41, D1040–6 (2013). 23203888 10.1093/nar/gks1215 PMC3531205

[b39] Durbin A. P. . Phenotyping of peripheral blood mononuclear cells during acute dengue illness demonstrates infection and increased activation of monocytes in severe cases compared to classic dengue fever. Virology 376, 429–435 (2008). 18452966 10.1016/j.virol.2008.03.028 PMC2546568

[b40] Rodenhuis-Zybert I. A. . Immature dengue virus: a veiled pathogen? PLoS Pathog. 6, e1000718 (2010). 20062797 10.1371/journal.ppat.1000718 PMC2798752

[b41] Chen Y. C. & Wang S. Y. Activation of terminally differentiated human monocytes/macrophages by dengue virus: productive infection, hierarchical production of innate cytokines and chemokines, and the synergistic effect of lipopolysaccharide. J. Virol. 76, 9877–9887 (2002). 12208965 10.1128/JVI.76.19.9877-9887.2002 PMC136495

[b42] Tsai W. Y. . High-avidity and potently neutralizing cross-reactive human monoclonal antibodies derived from secondary dengue virus infection. J. Virol. 87, 12562–12575 (2013). 24027331 10.1128/JVI.00871-13 PMC3838129

[b43] Diamond M. S. & Harris E. Interferon inhibits dengue virus infection by preventing translation of viral RNA through a PKR-independent mechanism. Virology 289, 297–311 (2001). 11689052 10.1006/viro.2001.1114

[b44] Ayala-Nunez N. V., Jarupathirun P., Kaptein S. J., Neyts J. & Smit J. M. Antibody-dependent enhancement of dengue virus infection is inhibited by SA-17, a doxorubicin derivative. Antiviral Res. 100, 238–245 (2013). 23994499 10.1016/j.antiviral.2013.08.013

[b45] Smit J. M., Bittman R. & Wilschut J. Low-pH-dependent fusion of Sindbis virus with receptor-free cholesterol- and sphingolipid-containing liposomes. J. Virol. 73, 8476–8484 (1999). 10482600 10.1128/jvi.73.10.8476-8484.1999 PMC112867

[b46] van Duijl-Richter M. K., Blijleven J. S., van Oijen A. M. & Smit J. M. Chikungunya virus fusion properties elucidated by single-particle and bulk approaches. J. Gen. Virol. 96, 2122–2132 (2015). 25872739 10.1099/vir.0.000144

[b47] Moesker B., Rodenhuis-Zybert I. A., Meijerhof T., Wilschut J. & Smit J. M. Characterization of the functional requirements of West Nile virus membrane fusion. J. Gen. Virol. 91, 389–393 (2010). 19828760 10.1099/vir.0.015255-0

[b48] Wong K. L. . Susceptibility and response of human blood monocyte subsets to primary dengue virus infection. PLoS One 7, e36435 (2012). 22574162 10.1371/journal.pone.0036435 PMC3344872

[b49] Trottier M. D. Jr., Palian B. M. & Reiss C. S. VSV replication in neurons is inhibited by type I IFN at multiple stages of infection. Virology 333, 215–225 (2005). 15721356 10.1016/j.virol.2005.01.009

[b50] Desmyter J., Melnick J. L. & Rawls W. E. Defectiveness of interferon production and of rubella virus interference in a line of African green monkey kidney cells (Vero). J. Virol. 2, 955–961 (1968). 4302013 10.1128/jvi.2.10.955-961.1968 PMC375423

[b51] Rodriguez-Madoz J. R. . Inhibition of the type I interferon response in human dendritic cells by dengue virus infection requires a catalytically active NS2B3 complex. J. Virol. 84, 9760–9774 (2010). 20660196 10.1128/JVI.01051-10 PMC2937777

[b52] Kwissa M. . Dengue virus infection induces expansion of a CD14(+)CD16(+) monocyte population that stimulates plasmablast differentiation. Cell. Host Microbe 16, 115–127 (2014). 24981333 10.1016/j.chom.2014.06.001 PMC4116428

[b53] Chareonsirisuthigul T., Kalayanarooj S. & Ubol S. Dengue virus (DENV) antibody-dependent enhancement of infection upregulates the production of anti-inflammatory cytokines, but suppresses anti-DENV free radical and pro-inflammatory cytokine production, in THP-1 cells. J. Gen. Virol. 88, 365–375 (2007). 17251552 10.1099/vir.0.82537-0

[b54] Long H. T. . Patterns of gene transcript abundance in the blood of children with severe or uncomplicated dengue highlight differences in disease evolution and host response to dengue virus infection. J. Infect. Dis. 199, 537–546 (2009). 19138155 10.1086/596507 PMC4333209

[b55] Simmons C. P. . Patterns of host genome-wide gene transcript abundance in the peripheral blood of patients with acute dengue hemorrhagic fever. J. Infect. Dis. 195, 1097–1107 (2007). 17357045 10.1086/512162 PMC4042601

[b56] Popper S. J. . Temporal dynamics of the transcriptional response to dengue virus infection in Nicaraguan children. PLoS Negl Trop. Dis. 6, e1966 (2012). 23285306 10.1371/journal.pntd.0001966 PMC3527342

[b57] Rolph M. S., Zaid A., Rulli N. E. & Mahalingam S. Downregulation of Interferon-{beta} in Antibody-Dependent Enhancement of Dengue Viral Infections of Human Macrophages Is Dependent on Interleukin-6. J. Infect. Dis. 204, 489–491 (2011). 21742851 10.1093/infdis/jir271

[b58] Tsai T. T. . Antibody-dependent enhancement infection facilitates dengue virus-regulated signaling of IL-10 production in monocytes. PLoS Negl Trop. Dis. 8, e3320 (2014). 25412261 10.1371/journal.pntd.0003320 PMC4239119

[b59] Kwan W. H. . Dermal-type macrophages expressing CD209/DC-SIGN show inherent resistance to dengue virus growth. PLoS Negl Trop. Dis. 2, e311 (2008). 18827881 10.1371/journal.pntd.0000311 PMC2553280

[b60] Rothman A. L., Medin C. L., Friberg H. & Currier J. R. Immunopathogenesis Versus Protection in Dengue Virus Infections. Curr. Trop. Med. Rep. 1, 13–20 (2014). 24883262 10.1007/s40475-013-0009-0 PMC4036645

[b61] Modhiran N. . Dengue virus NS1 protein activates cells via Toll-like receptor 4 and disrupts endothelial cell monolayer integrity. Sci. Transl. Med. 7, 304ra142 (2015). 10.1126/scitranslmed.aaa3863 26355031

[b62] Beatty P. R. . Dengue virus NS1 triggers endothelial permeability and vascular leak that is prevented by NS1 vaccination. Sci. Transl. Med. 7, 304ra141 (2015). 10.1126/scitranslmed.aaa3787 26355030

[b63] Zaitseva E., Yang S. T., Melikov K., Pourmal S. & Chernomordik L. V. Dengue virus ensures its fusion in late endosomes using compartment-specific lipids. PLoS Pathog. 6, e1001131 (2010). 20949067 10.1371/journal.ppat.1001131 PMC2951369

[b64] Mellman I. The importance of being acid: the role of acidification in intracellular membrane traffic. J. Exp. Biol. 172, 39–45 (1992). 1491231 10.1242/jeb.172.1.39

