# Supplementary material for: Antibody-Dependent Enhancement of Dengue Virus Infection in Primary Human Macrophages; Balancing Higher Fusion against Antiviral Responses
Source: Sci Rep. 2016 Jul 6;6:29201. doi: 10.1038/srep29201 (PMC4933910; doi:10.1038/srep29201)
Supplement: Supplementary Information [file srep29201-s1.pdf]

Title: Antibody-Dependent Enhancement of Dengue Virus Infection in Primary Human Macrophages; Balancing Higher Fusion against Antiviral Responses

Short title: Dengue ADE of Macrophages: balancing Fusion *vs* IFN.

Authors: Jacky Flipse[1], Mayra A. Diosa-Toro[1,2], Tabitha E. Hoornweg[1], Denise P.I. van de Pol[1], Silvio Urcuqui-Inchima[2], Jolanda M. Smit[1 #]

[1] Department of Medical Microbiology, University Medical Center Groningen, University of Groningen, the Netherlands
[2] Grupo Immunovirología, Facultad de Medicina, Universidad de Antioquia UdeA, Medellín, Colombia

# Corresponding author: Jolanda M. Smit
Department of Medical Microbiology, University Medical Center Groningen, The Netherlands
PO Box 30.001, EB88
9700 RB Groningen, The Netherlands.
E-mail: Jolanda.smit@umcg.nl
Phone number: 31-50-3632738
fax number: 31-50-3638171

Supplemental information

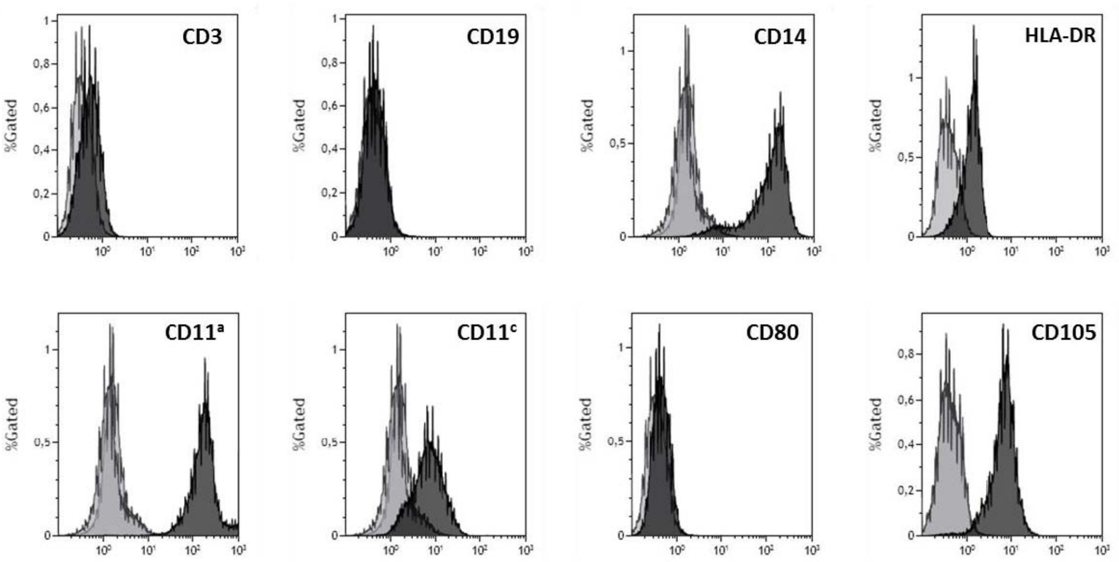

Fig A1 A representative phenotypic analysis of the primary human macrophages used in this study. Human Macrophages were generated in vitro by culturing primary monocytes for 6 days in the presence of M-CSF. Flow cytometric analysis shows a typical M-CSF-driven macrophage phenotype of: $CD3^-$, $CD19^-$, $CD14^{++}$, HLA-DR^+, $CD11a^+$, $CD11c^+$ $CD80^-$, and $CD105^+$. Light fill: isotype, dark fill: antibody.

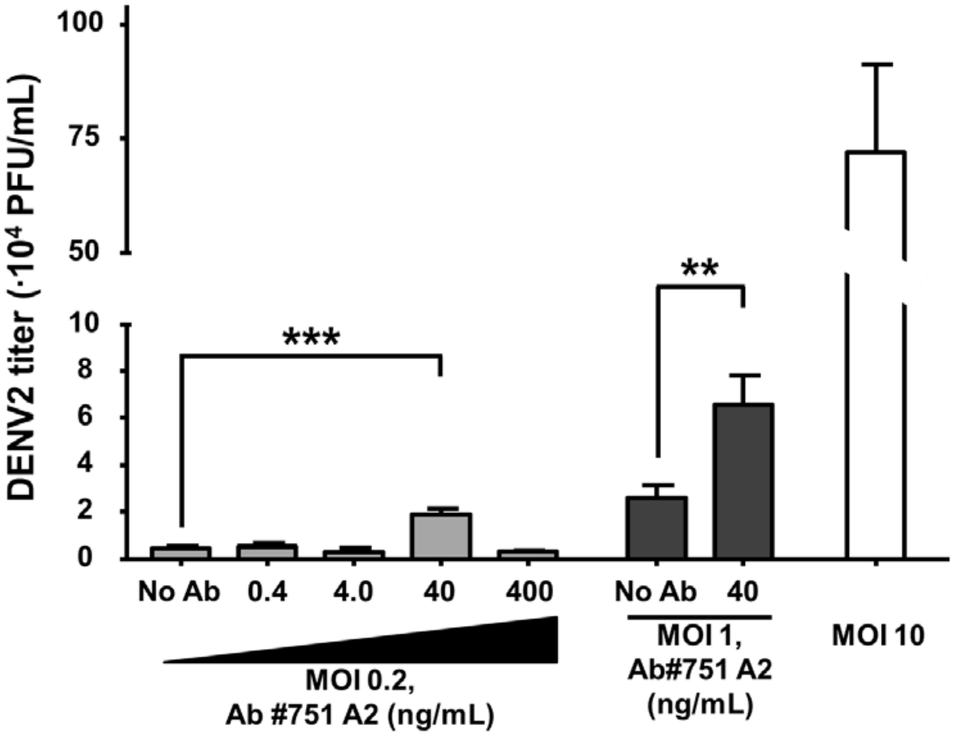

Fig A2 DENV production by primary human macrophages is MOI-dependent. Yet, the power of ADE is MOI-independent.

DENV2 was pre-incubated for 1h with increasing concentrations of monoclonal human antibody #751 A2 prior to infecting macrophages at MOI 0.2 (light grey bars), MOI 1 (dark grey) or MOI 10 (white bar). Shown is a representative donor of two experiments carried out in duplicate. P-value ≤0.01 (**) or ≤0.001 (***) as determined by 2-sided t-test.

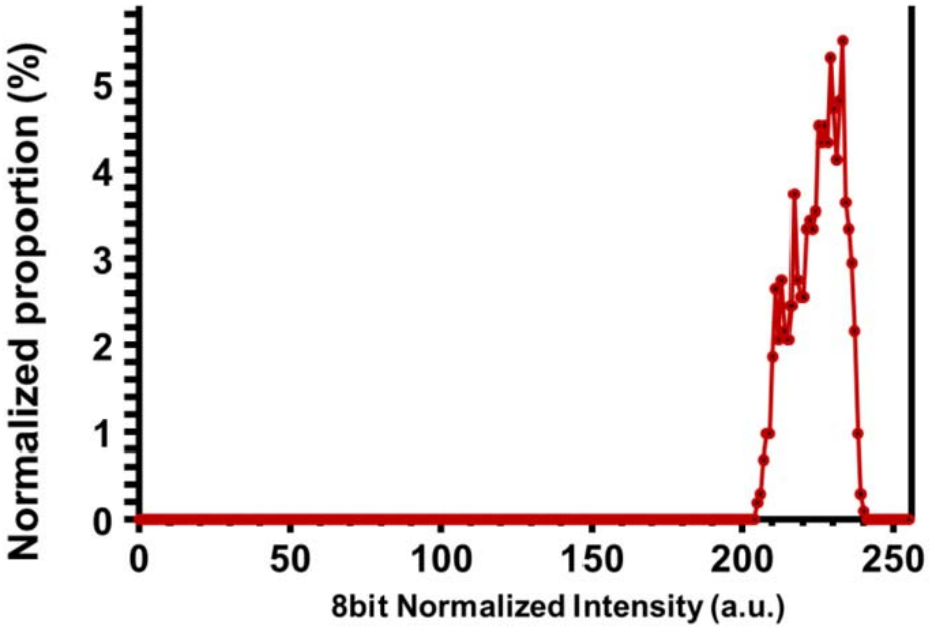

Fig A3 Controls to Fig 3B. Efficient and homogeneous labelling of DENV2 particles by PKH67. Purified DENV2 was labelled with PKH67 and the fluorescence intensity of the labelled was analysed by microscopy. The plot shows the percentage of particles for each intensity. The narrow peak indicates that the fluorescence intensity per particle was uniform.

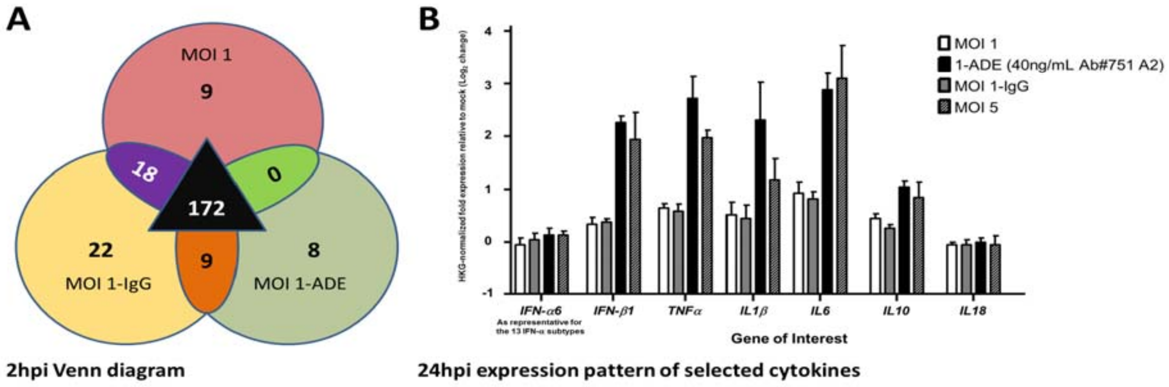

Fig A4 The presence of antibodies does not change the profile of primary macrophages infected with DENV.

(A) Primary human macrophages were infected with DENV at MOI 1, MOI 1-ADE and MOI 1-IgG. Total RNA was isolated at 2h and analysed by microarray. Genes whose expression had an averaged absolute fold change of at least 1.5-fold over the mock were selected. The Venn diagram is based on four donors.

(B) Expression of cytokine genes at 24h was calculated using the data derived from the microarray. Gene expression patterns were first normalized relative to five house-keeping genes (ActinB, Glucurondase beta, Hypoxanthine phosphoribosyltransferase 1, Heat shock protein 90 kDa alpha-beta 1, and glyceraldehyde-3-phosphate dehydrogenase). Subsequently, cytokines expression was calculated relative to the mock as Log_2-change. Shown is the SEM of 3 donors.

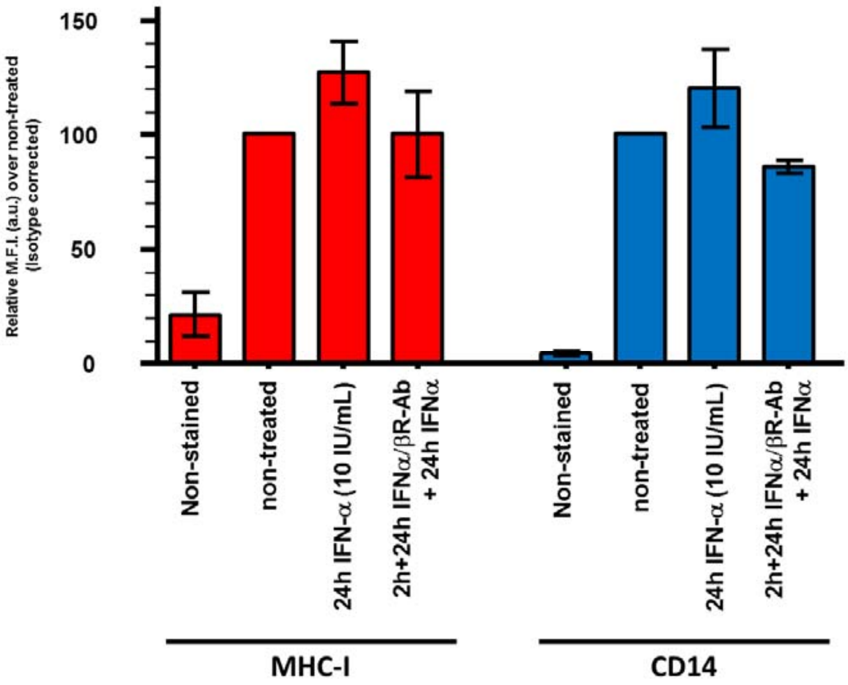

Fig A5 Specific blocking of the IFNαβR on primary human macrophages.

Macrophages were pre-incubated with the IFNα/βR-antibody prior to stimulation with 10 IU of IFNα. Surface expression of MHC-I (red) and CD14 (blue) were quantified at 24h by flow cytometry as the surface expression of these receptors depends, in part, on IFNαβR signalling(1-3). Shown is the SEM of 2 donors, normalized to non-treated conditions.

References

1. **Carotenuto, P., D. van Riel, A. Artsen, S. Bruijns, F. G. Uytdehaag, J. D. Laman, A. B. van Nunen, P. E. Zondervan, R. A. De Man, A. D. Osterhaus, and O. Pontesilli.** 2005. Antiviral treatment with alpha interferon up-regulates CD14 on liver macrophages and its soluble form in patients with chronic hepatitis B. Antimicrob. Agents Chemother. **49:**590-599. doi: 49/2/590 [pii].

2. **Park, C., S. Li, E. Cha, and C. Schindler.** 2000. Immune response in Stat2 knockout mice. Immunity. **13:**795-804. doi: S1074-7613(00)00077-7 [pii].

3. **Zhao, W., E. N. Cha, C. Lee, C. Y. Park, and C. Schindler.** 2007. Stat2-dependent regulation of MHC class II expression. J. Immunol. **179:**463-471. doi: 179/1/463 [pii].
